# The effect of job insecurity on knowledge hiding behavior: The mediation of psychological safety and the moderation of servant leadership

**DOI:** 10.3389/fpubh.2023.1108881

**Published:** 2023-03-13

**Authors:** Jeeyoon Jeong, Byung-Jik Kim, Julak Lee

**Affiliations:** ^1^College of Business, Korea University Business School, Korea University, Seoul, Republic of Korea; ^2^College of Business, University of Ulsan, Ulsan, Republic of Korea; ^3^Department of Psychology, Yonsei University, Seoul, Republic of Korea; ^4^Department of Industrial Security, Chung-Ang University, Seoul, Republic of Korea

**Keywords:** job insecurity, knowledge hiding behavior, psychological safety, servant leadership, moderated mediation model

## Abstract

As the global economy deteriorates because of the great shocks such as COVID-19 pandemic and wars among nations, the business environment is suffered from uncertainty and risk. To deal with it, several firms have attempted to maximize its efficiency *via* downsizing and restructuring to diminish costs. Thus, the degree of anxiety is increased among employees who worry about the loss of their job. The current research hypothesizes that job insecurity increases employees' knowledge hiding behavior by diminishing the degree of their psychological safety. In other words, psychological safety functions as the underlying process (i.e., mediator) in the job insecurity-knowledge hiding behavior link. Furthermore, this paper tries to examine the boundary condition of how to decrease the detrimental influence of job insecurity, focusing on the moderating effect of servant leadership. Utilizing a 3-wave time-lagged data from 365 Korean employees, we empirically demonstrated that employees who perceive job insecurity are less likely to perceive psychological safety, eventually increasing their knowledge hiding behavior. We also found that servant leadership functions as a positive moderator which buffers the negative impact of job insecurity on psychological safety. Theoretical and practical contributions are described.

## Introduction

Since the outbreak of the COVID-19 pandemic, the global economy has stagnated, resulting in many employees around the world losing their jobs (1, 2). In addition, as the robot automation system and artificial intelligence (AI) have advanced, employees have become more threatened with job loss and their job insecurity has worsened (3). Since job insecurity has a substantial adverse effect on both companies and their work force, organizational managers need to understand these effects and take timely action to prevent them (4–6). Existing studies on the topic show that job insecurity has a negative impact not only on organization members' attitudes and perceptions such as job satisfaction, organizational identification, and organizational commitment (7, 8), but also on their behaviors such as organizational deviance, safety behavior, innovative behavior, voice behavior, and organizational citizenship behavior (9–11). However, knowledge plays a pivotal role in an organization. Not only does it promote innovation in products, technologies, and services and help firms create value, but it also allows firms to secure competitive advantages in a rapidly changing competitive environment (12–14).

As described above, although existing studies have shed light on job insecurity's adverse effects, those have relatively overlooked certain important topics as follows. First, even though knowledge-management is very important to organizations/companies, to the best of our knowledge, few studies have examined the impact of job insecurity on “knowledge-related” behaviors, such as knowledge sharing or knowledge hiding behavior (3, 15). Of course, we acknowledge that existing contributions have explained the influence of job insecurity on several important employee behaviors such as in-role/extra-role behavior, safety behavior, voice behavior, innovative behavior, and organizational citizenship behavior (9–11, 15–20). However, the previous works have relatively underexplored the influence of job insecurity in the context of “knowledge”. Considering that knowledge creates value-added services and products, substantially affecting firms' competitive advantage (12–14), delving into the impact of job insecurity on employees' knowledge-related behaviors is highly recommended.

Second, “few” studies have examined the mediating mechanisms in the association between job insecurity and knowledge-related behaviors (3, 15, 21). These mechanisms merit scholarly attention because they would allow us to understand “why” job insecurity affects knowledge hiding and “what factors” strengthen or attenuate the effects of job insecurity in an organization (3, 15, 21).

Third, and most crucial, existing studies on the topic have overlooked the importance of leadership in attenuating the detrimental effects of job insecurity (3, 15). Most contributions have focused on the moderating role of individual characteristics (e.g., emotional intelligence, proactive personality, internal locus of control, and self-esteem) and organizational context (e.g., macro-economic conditions, social safety networks, and labor market insecurity)—i.e., how they reduce the adverse effects of job insecurity (6, 17, 22–25). Leaders have been known to substantially affect their followers' perceptions, attitudes, and behaviors by assigning tasks, assessing employees' performance, and making explicit and implicit rules (26, 27). Followers also perceive their leaders as main actors who symbolize the organizations they lead (28). Hence, it is useful to examine leadership's moderating effect on the consequences of job insecurity.

To open this “black box” as described above, our study explores the underlying mechanism (i.e., mediator) and its contingent variable (i.e., moderator) in the relationship between job insecurity and knowledge hiding behavior. Specifically, we suggest that an employee's psychological safety may mediate the relationship between job insecurity and knowledge hiding behavior. Moreover, servant leadership would positively moderate the association between job insecurity and psychological safety by buffering the negative effects of job insecurity. With this focus, our study extends existing knowledge about “why” job insecurity influences knowledge hiding behavior and “when” the impact of job insecurity changes.

## Theory and hypotheses

### Job insecurity and knowledge hiding behavior

First, this study suggests that job insecurity would increase the extent to which an employee hides knowledge (12, 15, 29). Knowledge hiding is defined as the deliberate concealment of knowledge when another employee requests information. Knowledge hiding makes it difficult to maintain an organization's competitive advantage and achieve success in a dynamic and rapidly changing organizational environment, because it prevents employees from sharing and transferring crucial work-related information, knowledge, and expertise (12–14). Although existing works have paid little attention to the association between job insecurity and knowledge hiding (15, 30), we rely on the conservation of resources theory (29) to suggest that job insecurity may increase the extent to which an employee hides knowledge. According to the conservation of resources theory (29), when an individual member faces the threat of losing resources, he or she is likely to attempt to reduce his or her energies and resources in the context or environment around him or her. Therefore, when an employee gets a sense of job insecurity, he or she is likely to redirect his or her energies and resources away from his or her tasks at work (15, 29). As a result, the employee would not make sufficient effort to share his or her knowledge with his or her colleagues.

***Hypothesis 1:*** An employee's job insecurity may increase their knowledge hiding behavior.

### Job insecurity and psychological safety

In this paper, we expect job insecurity to reduce employees' psychological safety (3, 31–33). Psychological safety refers to “an individual's perception of being able to show himself or herself without fear of adversely affecting his or her status, career, or self-image” ((33), p. 708). Although research on the relationship between job insecurity and employees' psychological safety is scarce (3, 32), it is obvious that job insecurity has a detrimental effect on employees' psychological safety (31, 33). When employees find an organization psychologically safe, they engage in risk-taking behaviors and have less fear that their opinions or ideas will be rejected, which makes them voice these ideas and opinions (31). Consequently, employees do not hesitate to seek their coworkers or supervisors for support and feedback, because they believe that the latter will not treat them unfavorably. On the contrary, when employees consider an organization psychologically unsafe, they will feel pressured and afraid and will find it difficult to freely express their opinions and thoughts (31, 33, 34). This can further decrease their psychological safety. Thus, organizations with high job insecurity will have employees feel that they are not being respected and protected by their employer, which is likely to prevent them from raising issues and opinions beneficial to the organization's success and development (21, 35–37).

***Hypothesis 2:*** An employee's job insecurity may reduce their psychological safety.

### Psychological safety and knowledge hiding behavior

In this study, we suggest that lower levels of psychological safety would increase the extent of an employee's knowledge hiding (31, 33, 38). An employee who feels a low level of psychological safety is not likely to cooperate with his or her colleagues, nor actively share his or her opinions and create knowledge (39–41). Thus, when his or her degree of psychological safety is low, the employee will be reluctant to share ideas, thoughts, and feelings with his or her colleagues and he or she will likely find it difficult to ask for help due to the fear or anxiety of being criticized (42). Additionally, a low degree of psychological safety may lead the employee to form negative perceptions and be skeptical of interpersonal relationships within the organization. As a result, the employee is likely to perceive his or her colleagues as rivals for survival in this environment (38, 43). Therefore, employees with low levels of psychological safety would abstain from sharing their knowledge in their organizations.

***Hypothesis 3:*** Decreased employees' psychological safety may increase their knowledge hiding behavior.

### Mediating role of psychological safety in the job insecurity-knowledge hiding behavior link

Integrating the dynamics discussed above (i.e., job insecurity, psychological safety, and knowledge hiding), we suggest that employees' psychological safety will mediate the relationship between job insecurity and knowledge hiding. Our mediation model can be supported by a context-attitude-behavior framework (44, 45). According to this perspective, an organization is characterized by a number of environmental or contextual factors, such as systems, practices, rules, and climates, which mold employees' attitudes and behaviors. In employees' minds, job insecurity is a critical context that influences their attitudes, such as psychological safety, and eventually affects their behaviors, such as knowledge hiding. Thus, we suggest that psychological safety mediates the relationship between job insecurity and knowledge hiding.

***Hypothesis 4:*** Employees' psychological safety may mediate the relationship between job insecurity and knowledge hiding behavior.

### Moderating effect of servant leadership in the job insecurity-psychological safety link

Moreover, and more important, we suggest that servant leadership would positively moderate the relationship between Job insecurity and psychological safety (23, 46, 47). In other words, our research sets boundary conditions by focusing on servant leadership and its role in the relationship between job insecurity and psychological safety. Our argument that job insecurity may lower employees' psychological safety is reasonable and acceptable. However, job insecurity may not always affect psychological safety in the same way because in real organizations, several contextual/contingent factors (e.g., personality, gender, age, leadership style, organizational climate, rule, and systems) moderate the relationship between the two variables (23, 46, 47).

This paper focuses on servant leadership, which is one of many leadership styles. This concept can be defined as “an (1) other-oriented approach to leadership (2) manifested through one-on-one prioritizing of follower individual needs and interests, (3) and outward reorienting of their concern for self toward concern for others within the organization and the larger community” [(48), p. 114]. In particular, we suggest that servant leadership mitigates job insecurity's detrimental effect on psychological safety. Servant leadership would provide followers with effective guidance about how to properly cope with any negative emotions, personal problems, and crises that stem from job insecurity (49–53). This leadership style encourages employees to feel a sense of respect, mutual trust, support, and self-worth in the organization, eventually reducing job insecurity's negative impact on psychological safety (49, 50, 53). For instance, when a leader's servant leadership is high, his or her behavior helps followers effectively resolve their anxiety and fear of unstable employment, even if they feel very insecure in their jobs. As a result, the employee is less likely to feel less safe psychologically (48, 50, 53).

In contrast, when a leader's servant leadership is low, employees find it difficult to cope with the negative emotions, personal issues, and crises concomitant to their unstable jobs. They are also likely to feel less respected and supported by their leader (49, 50, 53). Thus, leaders' low servant leadership may induce the followers who suffer in their unstable jobs to feel that they cannot effectively cope with said jobs and to become isolated from the organization. The negative influence of job insecurity on psychological safety would thus fail to become adequately resolved and may even be amplified (48, 50, 53).

***Hypothesis 5:*** Servant leadership may positively moderate the relationship between job insecurity and psychological safety.

**Figure 1:**
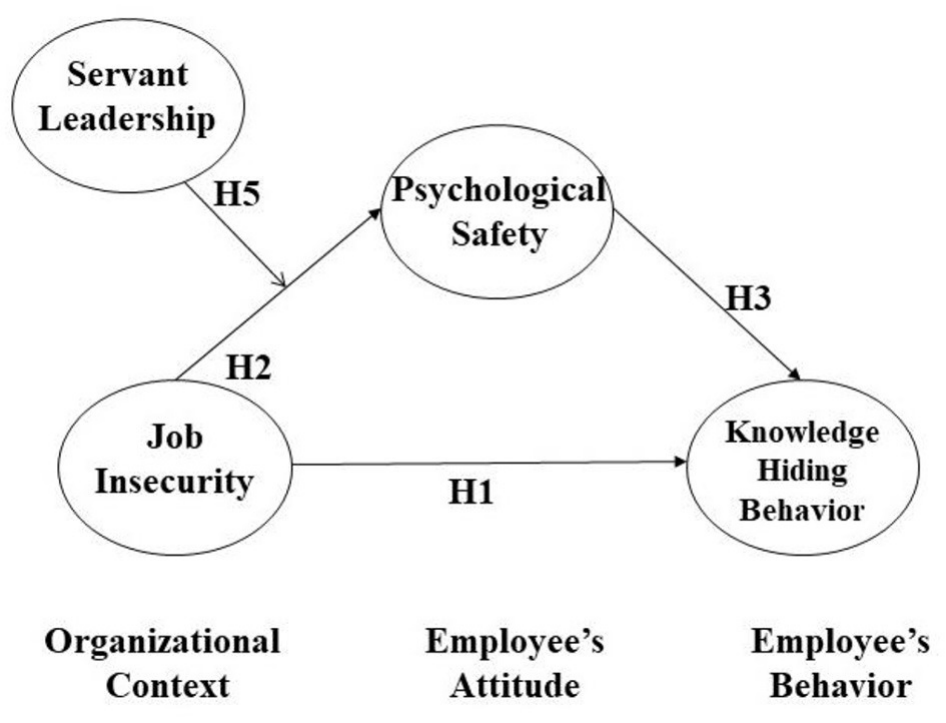
Theoretical model.

In sum, this study aims to understand how job insecurity influences knowledge hiding through the mechanism of employees' psychological safety. It predicts that servant leadership plays a moderating role in the link between job insecurity and psychological safety. Specifically, when servant leadership is high, job insecurity's negative impact is more modest than when it is low. We use structural equation modeling (SEM) to empirically test our proposed moderated mediation model. Our study contributes to and extends our knowledge on job insecurity in the following way. First, we focus on a knowledge-related variable (i.e., employees' knowledge hiding) that is affected by job insecurity. Second, we establish the underlying mechanism that links employees' job insecurity and knowledge hiding. Third, we propose a way in which organizations can attenuate the negative impact of job insecurity on employees' psychological safety by suggesting that servant leadership moderates the relationship between the two. Finally, we utilized three-wave time-lagged data, which minimizes the possibility of common method bias compared to a cross-sectional study. Figure 1 visualizes our hypothesized model.

## Methods

### Participants and procedure

We collected time-lagged data from three waves of surveys, with each new wave taking place 4 or 5 weeks after its predecessor. We used an online survey administration firm, which maintains approximately 3,450,000 panel data representatives of the Korean population, to recruit adults who work full time. We aim to minimize the common method variance problems inherent to cross-sectional studies by using 3-wave time-lagged data. The survey participants registered through an authentication system and had to identify their occupation status by providing an e-mail address or a mobile phone number. Existing research has established that using such an online survey system is a reliable way of obtaining diverse samples (54).

The operation function of the online system allowed us to ensure that the surveys were distributed to the same participants, with each new wave taking place 4 or 5 weeks after its predecessor. The survey system gave the participants sufficient time to respond to each survey (e.g., 2 or 3 days) and the respondents were allowed to answer the survey whenever they wanted.

In an effort to ensure an efficient response, the survey firm used traps and timestamps for geographic IP violators to monitor the integrity of the data. These tools prevented participants from repeatedly accessing the survey and completing the questionnaire. In order to guarantee the spontaneity and confidentiality of our participants' responses, the experts at the survey firm contacted them directly to request their consent to take part in our survey. We promised compliance with common ethical standards (such as ensuring anonymity) to those who agreed to participate, and received their informed consent. Our respondents received a cash reward equivalent to US$8. Our study received institutional review board (IRB) approval from our respective universities in South Korea.

A total of 1, 512 participants completed our first (Time 1) survey, designed to measure job insecurity and servant leadership. Four weeks later, 421 participants completed our second (Time 2) questionnaire, which measured employees' psychological safety. Five weeks later, the same participants received our third (Time 3) survey, which measured knowledge hiding. Thus, after excluding the missing data, out of 512 participants, our final analysis sample consisted of 365 participants who answered all three questionnaires (a response rate of 71.29%). We used several recommendations proposed by earlier studies to calculate our sample size. First, we used G^*^Power version 3.1.9.7 to determine the minimum sample size in order to assess whether our sample size was appropriate. According to our power analysis, which is based on previous research (55), 365 different samples had the adequate power (0.80) to identify a medium effect at an alpha level of *p* = 0.05. Furthermore, our research model followed the rule of ten (56), which states that one observable variable should include at least 10 cases. Considering that our research model contained 24 observable variables, thus our 365 cases would be sufficient. Table 1 provides a description of the characteristics of our respondents.

**Table 1:** Descriptive characteristics of the sample.

**Characteristic**	**Percent (%)**
**Gender**	
Male	52.1
Female	47.9
**Age (years)**	
20–29	14.0
30–39	36.1
40–49	33.5
50–59	16.4
**Education**	
Below high school	8.2
Community college	18.9
Bachelor's degree	61.1
Master's degree or higher	11.8
**Occupation**	
Office worker	71.2
Profession (Practitioner)	7.9
Manufacturing/Engineering	6.0
Public official	5.5
Sales and marketing	4.1
Administrative positions	3.8
Education	0.3
Freelancer	0.3
Others	0.9
**Position**	
Staff	22.7
Assistant manager	21.6
Manager or deputy general manager	33.4
Department/general manager or director and above	22.2
**Tenure (years)**	
Below 5	46.8
5–10	26.9
11–15	13.1
16–20	8.0
21–25	1.4
Above 26	3.8
**Industry type**	
Manufacturing	24.7
Wholesale/Retail business	11.8
Construction	11.5
Health and welfare	10.7
Information services and telecommunications	8.8
Education	7.9
Services	6.6
Financial/insurance	3.3
Consulting and advertising	1.1
Others	12.6

### Measures

We measured different variables at each of the three survey points. At time 1, we measured the degree of job insecurity and servant leadership. At time 2, employees were asked to report their psychological safety. Finally, at time 3, we measured the employees' knowledge hiding behavior from their direct supervisors. All variables were measured on five-point Likert scales (1 = strongly disagree, 5 = strongly agree). We also calculated the variables' internal consistency *via* their Cronbach alpha values.

### Job insecurity (Time 1, collected from employees)

We measured employees' job insecurity using five items developed by Kraimer et al. (57). A sample item read: “My job is not a secure one.” The value of the Cronbach's alpha is 0.91.

### Servant leadership (Time 1, collected by employees)

Servant leadership was measured by Liden et al. (58) seven items. A sample item is: “My supervisor puts my best interests ahead of his/her own”. The value of Cronbach's alpha is 0.79.

### Psychological safety (Time 2, collected from employees)

We measured employees' psychological safety using seven items (31). A sample item read: “I am able to bring up problems and tough issues in this organization.” The value of the Cronbach alpha is 0.80.

### Knowledge hiding behavior (Time 3, collected from employees' direct supervisors)

We utilized five items of knowledge hiding behavior scale which consists of eleven items (59). Each employee's immediate supervisor evaluated the level of his or her knowledge hiding behavior. The reason why we shortened the full items is that the five items were validated by existing empirical research which were conducted in the context of South Korea (60). A sample item is “This employee pretended that he or she couldn't find the information that his or her colleagues wanted”, and “Thie employee gives colleagues a little bit of assistance, but didn't help them to the extent they wanted”. The value of the Cronbach's alpha is 0.95.

### Control variables

We measured several control variables in addition to our primary variables of interest. In line with existing research on knowledge hiding (59), we used participants' gender, tenure, and education level, all measured in our Time 2 survey.

### Statistical analysis

We first conducted a frequency analysis to confirm the demographic characteristics of our participants. The relationships among the variables in our study were calculated through correlation analysis using SPSS 26 and Pearson. We followed Anderson and Gerbing (61) by adopting a two-step procedure (i.e., a measurement model and a structural model). We then conducted a series of confirmatory factor analyses (CFA) to evaluate the empirical distinctiveness of our main variables (i.e., job insecurity, servant leadership, psychological safety, and knowledge hiding). Afterwards, we used AMOS 23 to run our structural model and performed an analysis of moderated mediation using the maximum likelihood (ML) estimator.

We calculated the comparative fit index (CFI), the Tucker-Lewis index (TLI), and the root mean square error of approximation (RMSEA) to ensure the empirical distinctiveness of each of our main variables. Browne and Cudeck (62) suggest that it is ideal to have CFI and TLI values above 0.90 and an RMSEA value below 0.06. Next, we ran a bootstrapping analysis to confirm the significance of the indirect effect of psychological safety (63). Finally, we performed a bootstrapping analysis by estimating a 95% confidence interval (CI) to see if our hypothesis of mediation and indirect mediation was supported. When the confidence interval (CI) excludes zero (0), the indirect effect is significant at level 0.05 (63).

## Results

### Descriptive statistics

We find that some of the variables in our study are significantly correlated with one another (e.g., job insecurity, servant leadership, psychological safety, and knowledge hiding). Table 2 presents the descriptive statistics of and the correlations between our variables.

**Table 2:** Correlation between research variables.

	**Mean**	**S.D**.	**1**	**2**	**3**	**4**	**5**	**6**	**7**
1. Gender_T2	1.48	0.50	–						
2. Education_T2	2.76	0.76	−0.14^**^	–					
3. Tenure_T2	7.49	7.26	−0.24^**^	0.01	–				
4. Position_T2	2.96	1.60	−0.39^**^	0.24^**^	0.28^**^	–			
5. Job insecurity_T1	2.79	0.87	−0.06	−0.07	0.01	0.11^*^	–		
6. SL_T1	3.07	0.67	−0.11^*^	0.05	0.03	0.15^*^	−0.00	–	
7. PS_T2	3.20	0.60	−0.17^**^	0.10	0.12^*^	0.19^**^	−0.26^**^	0.39^**^	–
8. KHB_T3	2.23	0.84	0.09	−0.10	0.09	0.03	0.23^**^	0.03	−0.22^**^

^*^p < 0.05, ^**^p < 0.01. S.D. means standard deviation, SL means servant leadership, PS means psychological safety, and KHB indicates knowledge hiding behavior. As for gender, males are coded as 1 and females as 2. As for position, general manager or higher are coded as 5, deputy general manager and department manager 4, assistant manager 3, clerk 2, and others below clerk as 1. As for education, “below high school diploma” level is coded as 1, “community college” level as 2, “bachelor's” level as 3, and “master's degree or more” level is coded as 5.

### Measurement model

We first conduct a series of CFAs to ensure that each of our main variables is empirically distinctive (job insecurity, servant leadership, psychological safety, and knowledge hiding). Specifically, the chi-square difference test allows us to establish whether our proposed four-factor model (job insecurity, psychological safety, servant leadership, and knowledge hiding) is better than alternative models (e.g., a three-factor model, a two-factor model, and a one-factor model).

First, the hypothesized 4-factor model had a good and acceptable fit (χ^2^ (df = 109) = 212.224; CFI = 0.974; TLI = 0.967; RMSEA = 0.051). Then, we conducted a series of chi-square difference tests by comparing the 4-factor model with a 3-factor model (χ^2^ (df = 112) = 1239.387; CFI = 0.715; TLI = 0.655; RMSEA = 0.165), a 2-factor model (χ^2^ (df = 114) = 1699.100; CFI = 0.600; TLI = 0.523; RMSEA = 0.194), and a 1-factor model (χ^2^ (df = 115) = 1763.298; CFI = 0.584; TLI = 0.508; RMSEA = 0.197). The results of the chi-square difference tests showed that the 4-factor model was better than others. Thus, this result means that our four research variables have an appropriate degree of discriminant validity.

### Structural model

Our study uses a moderated mediation model that adds mediators and moderators to the relationship between job insecurity and knowledge hiding. First, the mediator (psychological safety) mediates the relationship between job insecurity and knowledge hiding. Second, the moderator (servant leadership) ameliorates the negative relationship between job insecurity and psychological safety.

Next, we multiply job insecurity and servant leadership in the moderation structure to create an interaction term. The variables are mean-centered to avoid multicollinearity. This technique reduces the multicollinearity and the correlation between our two variables (64).

We calculate tolerance values and variance inflation factors (VIF) to assess the effects of multicollinearity (64). We find that the tolerance values of job insecurity and servant leadership are 1.000 and 1.000, respectively. Their VIF values are 1.001 and 1.001, respectively. The results demonstrate/suggest that the two variables (job insecurity and servant leadership) are free of multicollinearity, as their tolerance values are >0.2 and their VIF values are < 10.

### Results of the mediation analysis

We conduct a chi-square difference test that compares our full mediation model to a partial mediation model to identify the best mediation model. Except for the direct path from job insecurity to knowledge hiding behavior, the full and the partial mediation models are identical. The results of the fit indices are reasonable both for the full mediation model [χ^2^ = 295.377 (df = 137), CFI = 0.957, TLI = 0.947, and RMSEA = 0.056] and for the partial mediation model [χ^2^ = 287.111 (df = 136), CFI = 0.959, TLI = 0.949, and RMSEA = 0.055). Yet, the chi-square difference test between the two (Δχ^2^ [1] = 8.266, *p* < 0.01) indicates that the partial mediation model is significantly better than the full mediation model. The findings suggest that job insecurity affects knowledge hiding behavior both “indirectly” (through psychological safety) and “directly”.

We add our control variables into our research model since the variables can affect the dependent variable (i.e., knowledge hiding behavior). And we found that all control variables are statistically insignificant.

Then, the results showed that job insecurity and knowledge hiding are positively and significantly correlated (β = 0.17, *p* < 0.01). Thus, Hypothesis 1 is supported. Hypothesis 1 expected the “partial” mediation model (which was superior to full mediation) to contain the coefficient value of the relationship between job insecurity and knowledge hiding behavior. These results are consistent with the fact that the partial mediation model's fit indices are superior to those of the full mediation model. When we take the two together, we accept the Hypothesis 1. That is, rather than only indirectly, job insecurity is more likely to both “directly” and “indirectly” affect knowledge hiding behavior *via* psychological safety.

**Figure 2:**
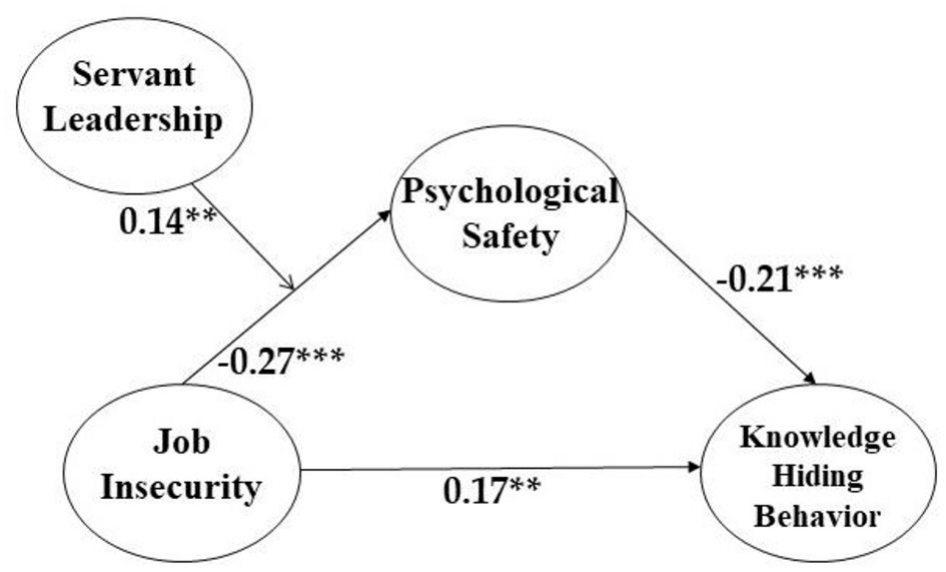
Coefficient values of our research model (**p < 0.01, ***p < 0.001. All values are standardized).

We also discovered a significant negative association between job insecurity and psychological safety (β = −0.27, *p* < 0.001), supporting Hypothesis 2. In addition, Figure 2 indicates that psychological safety is negatively and significantly correlated with knowledge hiding behavior (β = −0.21, *p* < 0.001), supporting Hypothesis 3 (Please see Table 3).

**Table 3:** Results of structural model.

**Hypothesis**	**Path (Relationship)**	**Unstandardized estimate**	**S.E**.	**Standardized estimate**	**Supported**
1	Job insecurity-> Knowledge Hiding Behavior	0.155	0.053	0.165^**^	Yes
2	Job insecurity –> Psychological Safety	−0.145	0.030	−0.267^***^	Yes
3	Psychological Safety -> Knowledge Hiding Behavior	−0.358	0.102	−0.208^***^	Yes
5	Job insecurity × Servant Leadership	0.099	0.037	0.137^**^	Yes

^*^p < 0.05, ^**^p < 0.01, ^***^p < 0.001. Estimate indicates standardized coefficients. S.E. means standard error. The coefficient value of the path from job insecurity to knowledge hiding behavior (H1) was in the partial mediation model which was not accepted as a final model.

### Bootstrapping

Hypothesis 4 predicted that psychological safety mediates the impact of job insecurity on knowledge hiding. To test Hypothesis 4, we perform a bootstrapping analysis (sample size = 10,000) (63). We use the resulting bias-corrected confidence interval (CI) to determine whether the mediation is significant. The 95% confidence interval (CI) should exclude the zero for us to be able to declare the mediation significant (63). We use these guidelines and a sample of 10,000 to confirm that psychological safety's indirect effect is significant, as the confidence interval does not contain zero (95% confidence interval [0.020, 0.105]. Thus, the mediating effect of psychological safety is statistically significant, and Hypothesis 4 receives some support. Table 4 illustrates the direct, the indirect, and the total effect of job insecurity on knowledge hiding.

**Table 4:** Direct, indirect, and total effects of the final research model.

**Model (Hypothesis 4)**	**Direct effect**	**Indirect effect**	**Total effect**
Job insecurity -> Psychological Safety -> Knowledge Hiding Behavior	0.165	0.056	0.221

All values are standardized.

### Result of the moderation analysis

**Figure 3:**
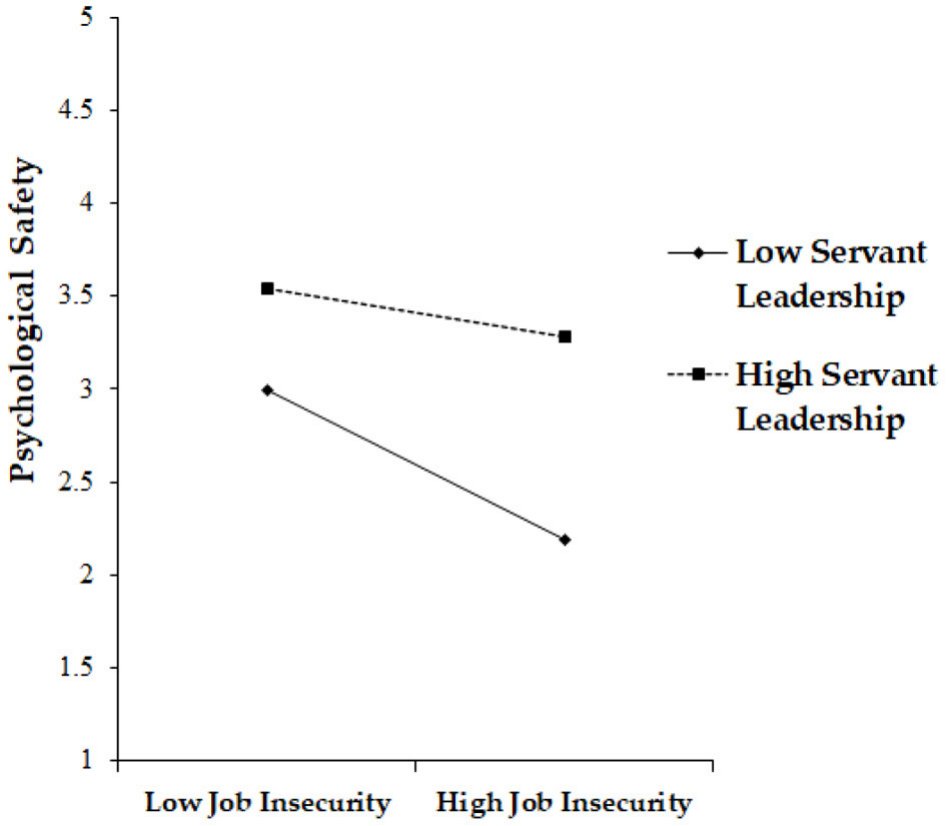
Moderating effect of servant leadership in the job insecurity–psychological safety link.

Hypothesis 5 proposed that servant leadership positively moderates the negative relationship between job insecurity and psychological safety. To test Hypothesis 5, we mean-centered the two variables and generated an interaction term (i.e., job insecurity × servant leadership). The results demonstrated that the interaction term is significantly related to psychological safety (β = 0.14, *p* < 0.01). As expected, servant leadership plays a buffering role in the negative relationship between job insecurity and psychological safety. Specifically, the negative effect of job insecurity on psychological safety is buffered when servant leadership is high compared to when it is low. Taken together, these findings support Hypothesis 5 (Please see Figure 3).

## Discussion

We have examined and tested the mediating effect of psychological safety in the relationship between job insecurity and knowledge hiding, as well as the buffering role of servant leadership in the association between job insecurity and psychological safety. We use a three-wave time-lagged study and find that employees who feel job insecurity are less likely to feel psychological safety, which leads to increased knowledge hiding. We also establish that servant leadership functions as a buffering factor, which positively moderates the relationship between job insecurity and psychological safety. We can draw several theoretical and practical implications from our results.

### Theoretical implications

From a theoretical perspective, this study makes the following contributions. First, we have examined the relationship between job insecurity and knowledge-related behaviors. The relationships between job insecurity and behavioral outcomes (e.g., innovative behavior, voice behavior, organizational citizenship behavior, safety behavior, and counterproductive work behavior) are well-documented (15, 16, 18–20). However, surprisingly, the research on knowledge-related outcomes is considerably limited (3, 15). Given that knowledge is the driving force behind organizational innovation and ultimately determines firms' competitive advantage and success (12–14), it is necessary to examine the relationship between job insecurity and knowledge-related behaviors. Therefore, this study enriches the literature on job insecurity by engaging in this line of research.

Second, even though existing research has highlighted the need to explore additional underlying mechanisms and boundary conditions in the relationship between job insecurity and knowledge-related behaviors (3, 15), few studies have done so. To better understand why and when this relationship occurs, it is important to examine its mediators and moderators. By integrating a context-attitude-behavior framework with social identity theory, this study has emphasized the roles of employees' psychological safety as a mediating mechanism and servant leadership as a boundary condition. In doing so, we extend existing research on the relationship between job insecurity and knowledge hiding by adding a substantive intermediating mechanism and boundary condition to interpret how job insecurity affects knowledge hiding and when the impact of job insecurity is minimized or strengthened.

Third, existing research on job insecurity has established/discovered that leadership plays a critical role in buffering the negative impact of job insecurity (46). However, most studies have focused on individual-level variables, such as self-esteem, internal locus of control, proactive personality, psychological capital, resilience, and emotional intelligence (6, 17, 22–25), and or macro-level contextual moderators, such as labor market insecurity, social safety networks, and macro-economic conditions (3, 15). Previous studies (27, 28, 46) indicate that leadership is a critical factor in encouraging subordinates' perceptions of and attitudes and behaviors toward the organization. Thus, servant leadership functions as a pivotal contingent factor in the relationship between job insecurity and knowledge hiding *via* employees' psychological safety. Our moderated-mediation model highlights the essential role of servant leadership when examining the influence of job insecurity on knowledge hiding.

### Practical implications

The results of our study also have some practical implications. First, they show that job insecurity has important implications for knowledge hiding. Our SEM reveals that job insecurity leads to increased knowledge hiding. Organizational managers should remember that job insecurity hinders the flow of knowledge across organization members because knowledge is crucial to the achievement of firms' organizational success and competitive advantage. Thus, reducing employees' job insecurity might prove more effective in preventing knowledge hiding than material or financial incentives. Organizations could implement human resource management practices like mentoring programs, long-term contracting with employees, and fair performance evaluations (46) to encourage such reductions.

Second, we suggest that psychological safety mediates the relationship between job insecurity and knowledge hiding. Reduced psychological safety can increase job insecurity's influence on knowledge hiding behaviors. Thus, implementing specific measures to fortify employees' organizational identification should be a concern for managers. They could increase firm reputation through firm activities, systems, or lectures that inspire employees to identify themselves as members of their organizations and form positive organizational images in their minds. Therefore, managers should strive not only to decrease job insecurity but also to increase employees' psychological safety.

Third, we propose that servant leadership buffers the negative impact of job insecurity on psychological safety. In particular, in today's rapidly changing business environment, it is necessary to guide, facilitate, and inspire employees to help solve, and cope with, difficulties within their organizations, such as job insecurity. By encouraging leaders to engage in servant behaviors *via* training systems and courses (e.g., emphasizing the importance of subordinate guidance, discovering subordinates' potential and growth, providing subordinates with opportunities to maximize their abilities), leaders can develop a servant leadership style.

### Limitations and suggestions for future research

We believe that the current study may meaningfully contribute to the literature on job insecurity and knowledge hiding behavior, but it still has some limitations that need to be addressed. First, this research could not measure employees' job insecurity in an objective manner because it only uses self-reported survey data, which is subjective. However, objective indicators, such as the downsizing rate, may not directly influence employees' perceptions and attitudes because these objective characteristic (e.g., downsizing rate) tend to be interpreted through each individual's sense-making processes, which means that the objective measure would be unconsciously reflected in each employee's responses. Thus, this paper suggests that future research needs to not only use both subjective and objective measures, but also needs to compare the differential effects of these different measures. Second, this research could not properly consider a number of external factors that can substantially affect employees' job insecurity. Numerous objective factors affect an employee's perception on his or her subjective job insecurity, such as companies' downsizing rates, the quality or characteristics of the human resource management system in place, and the features of the social insecurity system at the national level (60). Therefore, we suggest that future research should more fully consider the issue by controlling for such objective variables.

Third, the fundamental values and spirit that servant leadership pursues may be universal in in the West and in the East (65, 66). However, a number of cultural differences do affect individuals' understandings of the role leadership plays, and these differences eventually affect employees' responses to different leadership styles. South Korea has been affected by the Confucian hierarchical systems for the past several centuries, so Korean employees may be more familiar with a culture of command and discipline than their Western counterparts (65). As a result, Korean employees are likely to feel that their leaders' servant behaviors are not natural and effective in a real organization. Therefore, the results of this study should be interpreted with care.

## Conclusion

Our study delved into the influence of job insecurity on employees' knowledge hiding behavior. The results demonstrated that job insecurity promotes employees' knowledge hiding behavior through the mediating role of psychological safety. In turn, servant leadership functions as a positive moderator in the relationship between job insecurity and the psychological safety. The results indicate that the level of employees' psychological safety is an underlying mechanism in translating job insecurity into individual knowledge hiding behavior. In addition, servant leadership plays a buffering role, which decreases the negative influence of job insecurity. Although this research has some limitations, we expect that it can positively contribute to not only expanding the literature on job insecurity from the theoretical perspective but also providing practical implications for leaders and practitioners in organizations.

## Data availability statement

The raw data supporting the conclusions of this article will be made available by the authors, without undue reservation.

## Ethics statement

The studies involving human/animal participants were reviewed and approved by Macromill Embrain Group of Ethics Committee. Macromill Embrain Group is the company providing market research service and their approval is sufficient according to the local requirements. The patients/participants provided their written informed consent to participate in this study.

## Author contributions

JJ contributed by generating a research idea, data analysis, and writing the original draft of the manuscript. B-JK contributed in data collection, methodology, review, and editing the manuscript. JL contributed in thoroughly revising, editing, and re-writing the manuscript. All authors have read and agreed to the published version of the manuscript.
